# Low-intensity focused ultrasound of the spine in the treatment of chronic pain and movement disorder: a scoping review

**DOI:** 10.3389/fpain.2025.1606672

**Published:** 2025-06-17

**Authors:** Kyung Seol, Brian Hong, Nicolas Kelhofer, Suguna Pappu, Michael Oelze

**Affiliations:** ^1^ Carle Illinois College of Medicine, University of Illinois at Urbana–Champaign, Champaign, IL, United States; ^2^ Department of Neurosurgery, Carle Foundation Hospital, Urbana, IL, United States; ^3^ Beckman Institute for Advanced Science and Technology, University of Illinois at Urbana–Champaign, IL, United States; ^4^ Department of Electrical and Computer Engineering, University of Illinois at Urbana–Champaign, IL, United States; ^5^ Department of Biomedical and Translation Sciences, Carle Illinois College of Medicine, University of Illinois at Urbana–Champaign, Champaign, IL, United States

**Keywords:** focused ultrasound (FUS), low intensity focused ultrasound (LIFU), chronic neuropathic pain, neuromodulation, noninvasive pain treatment, movement disorders, spinal cord, dorsal root ganglia (DRG)

## Abstract

Low-Intensity Focused Ultrasound Stimulation (LIFU) is a noninvasive and nondestructive neuromodulatory method with growing evidence for the safe and effective treatment of chronic pain. However, the effect of LIFU applied to the spine region, including the spinal cord and dorsal root ganglia, is not well understood. In this work, we review current advances in LIFU of the spine region for the treatment of chronic neuropathic pain and movement disorders to explore potential clinical applications and indicate a direction for future study. To assess the current state of LIFU application to pain modulation over the spinal cord region, a systematic search was performed according to PRISMA guidelines using PubMed, Web of Science, Scopus, and citation matching through December 17, 2024. Inclusion criteria were English language, non-tissue-damaging ultrasound neuromodulation, intervention over the spinal cord region, and relation to neuropathic pain. Exclusion criteria were existing review papers, extracorporeal shockwave therapy, tissue-destructive ultrasound treatments, non-focused ultrasound, and *in vitro* experiments. Preliminarily, title and abstract screening identified 15 studies, all using animal models. While results varied with different target sites and ultrasound parameters, LIFU was found to reduce allodynic response and suppress movement disorders such as spasticity and tremor. There are limited animal studies and no completed human clinical trials that analyze the effect of LIFU on spinal neural tissue. Further, there has not been a study that aims to optimize ultrasound parameters in the spine region or a thorough investigation correlating targets in the spinal regions to the desired outcome. We reviewed the current understanding of LIFU of the spine region for treating chronic pain, spasticity, and tremors to identify current advances and gaps in the literature. Our review highlights the need for further study in the efficacy and safety of LIFU applied to the spinal region of animals and humans, given the wide variation in sonication parameters, inconsistent treatment effects, and unexplored mechanisms of action.

## Introduction

Chronic pain affects over 20.4% of U.S. adults, imposing an annual economic burden of $560 billion (1). Among those affected, chronic neuropathic pain (CNP) impacts 33 million individuals (2), with limited noninvasive treatment options available.

Movement disorder (MD) can stem from dysfunction in the brain or spinal cord and is commonly classified into hypokinetic, hyperkinetic, and miscellaneous categories (3). MD can be a condition that is inherited or acquired secondary to a disease such as stroke and spinal cord injury. Spasticity is an MD that affects up to 38% of stroke survivors and 65% of individuals with spinal cord injuries (SCI) (4, 5). Unfortunately, medically refractory MD—such as spasticity and tremors—have few effective noninvasive treatment options (6).

**Figure 1:**
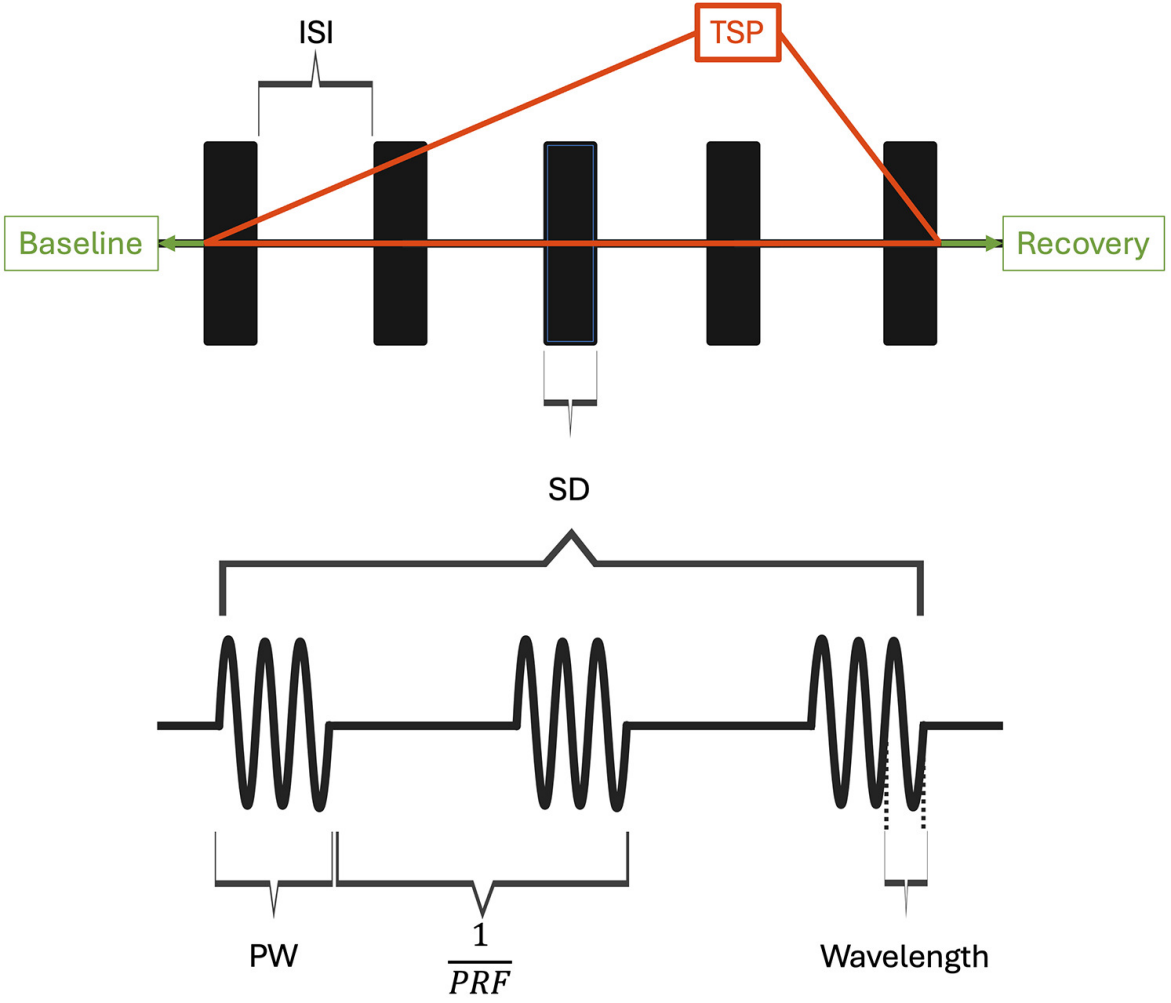
Ultrasound parameters and pulsing regime are visualized. ISI, inter-sonication interval; TSP, total sonication period; SD, sonication duration; PW, pulse width; PRF, pulse repetition frequency. Duty cycle (DC) is the product of pulse width and PRF. When DC is 1, FUS is applied continuously within the sonication duration. DC in this context is exclusively used to characterize parameters within the sonication parameters. Created with BioRender.com.

**Figure 2:**
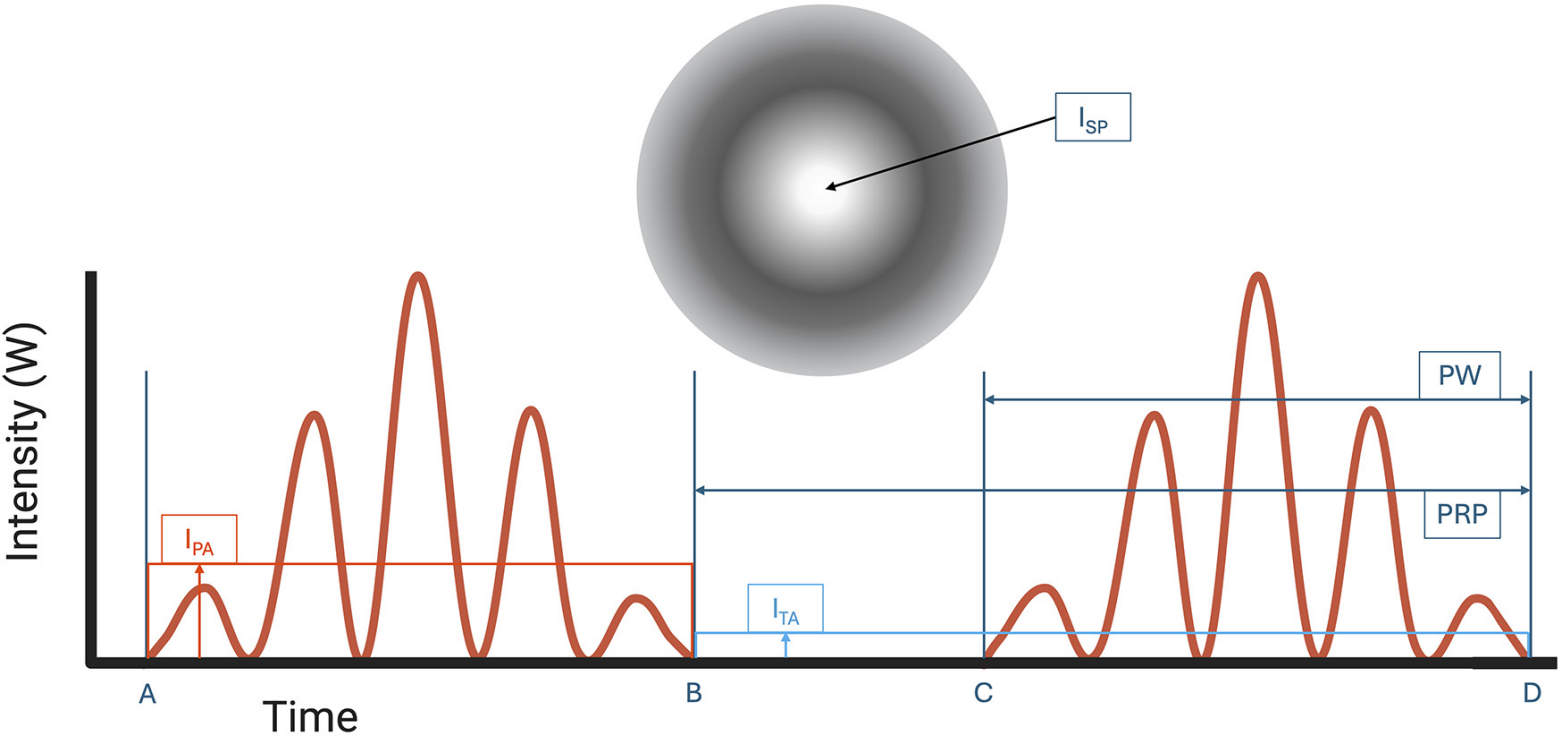
Pulse width (PW, time from A to B or C to D) and pulse repetition period (PRP, time from B to D) is shown. *I*_SPTA_ is calculated as the temporal average intensity (*I*_TA_) at the region of Spatial-peak intensity (*I*_SP_). *I*_SPTA_ is used to determine thermal safety profile of FUS. *I*_SPPA_ is calculated as the pulse average intensity (*I*_PA_) at the region of spatial-peak intensity (*I*_SP_) on the ultrasound intensity map. Note that *I*_TA_ is always lower than *I*_PA_ because *I*_TA_ includes intensities when resting between time B and C. Created with BioRender.com.

Focused ultrasound (FUS) has emerged as a novel therapeutic technology to treat a wide range of neurological conditions (7, 8). FUS works by converging ultrasound waves to a specific focal point in space determined by the transducer's geometry. High intensity focused ultrasound (HIFU) is clinically used to thermally ablate and lesion the ventral intermediate thalamus in patients with essential tremors, with ongoing studies in focal epilepsy and brain tumor treatment (9, 10). In contrast, low intensity focused ultrasound (LIFU) is a developing noninvasive and nondestructive FUS neuromodulation technology that employs mechanical sound waves to inhibit or stimulate neurons. In the application of LIFU, multiple exposure parameters, including the pulse repetition frequency (PRF), pulse width (PW), frequency of ultrasound, duty cycle (DC), and intensity as shown in Figures 1, 2, must be carefully optimized to ensure therapeutic efficacy while maintaining safety.

Currently, the exact mechanism of LIFU neuromodulation is unclear. Plaskin et al. (11, 12) proposed the neuronal intramembrane cavitation excitation (NICE) model whereby LIFU induces cell depolarization due to microcavitation within the cell membrane to create an action potential. A more recent hypothesis suggests the activation of mechanosensitive ion channels as the primary driver of the effect (13). Specific sonication parameters with LIFU can be used to create temporary openings in the blood-brain barrier (BBB). BBB opening is an active field of research primarily involving drug delivery (14). LIFU has been shown to create an online and offline physiological effect, which is observed when the FUS treatment is active and during the period after FUS treatment respectively, additionally suggesting a neuroplastic effect (15).

Recent preclinical studies of transcranial LIFU have shown promising effects in modulating pain and essential tremors. Kim et al. (16) found that LIFU can suppress pain hypersensitivity in a rodent model of sickle cell anemia. Riis et al. (17) showed that targeting the ventral intermediate nucleus (VIM) of the thalamus–a target of high intensity FUS thermal ablation for medically refractory essential tremor—with LIFU led to a reversible 98% tremor reduction without lasting tissue damage in rodents. Deveney et al. (18) reported clinically significant improvement among 10 participants following LIFU targeting of the VIM thalamus. There are further ongoing clinical trials of transcranial LIFU for chronic neuropathic pain (19–21).

Currently, clinical neuromodulation in the spinal region consists of epidural spinal cord stimulation (SCS), dorsal root ganglion (DRG) stimulation, and transcutaneous electrical nerve stimulation (TENS). In SCS and DRG stimulation, implanted electrodes deliver current to the spinal cord or DRG to inhibit the conduction of pain, with both methods being clinically validated for use in chronic pain conditions (22–25). These devices are believed to exert analgesic effects by electrically suppressing neurons in the spinothalamic tract and dorsal root ganglion (26, 27). However, both methods face complications with infection, lead migration, and hardware malfunction (25). TENS offers the advantage of being non-invasive, delivering electrical stimuli through patches at the skin surface. However, the efficacy of TENS for chronic pain management is still unclear and debated in the literature (28). While LIFU targeting the spinal region is less studied, with no clinical application reported to date, it is a promising technology that combines the non-invasiveness of TENS with the depth penetration and spatial resolution of more invasive techniques such as SCS.

The first evidence of spinal cord response to ultrasound was reported in the 1950s (29–31). In a frog model, sonication of the lumbar enlargement region induced hindlimb paralysis without thermal or cavitation-related effects. Despite early promise, research on nondestructive ultrasound remained limited until recently. Given the established therapeutic potential of spinal cord and dorsal root stimulation, LIFU neuromodulation presents a promising avenue for future clinical transition. This review examines the current preclinical data on the neuromodulation effect of LIFU on the spinal cord and DRG for the treatment of chronic pain and MD.

## Methods

### Search methodology

To assess the current state of LIFU application to pain modulation over the spinal cord region, a literature search was performed according to PRISMA 2020 guidelines using PubMed, Web of Science, Scopus, and citation matching through December 17, 2024. Search terms were adjusted based on the specific vocabulary of each indexed database, with search fields including title, abstract, and author keywords. After duplicate removal and subsequent title, abstract, and full-text screening by two independent reviewers, a total of 15 papers were retrieved. Given the purpose of this scoping review, the quality of the papers was not statistically investigated.

### Inclusion and exclusion criteria

To be included in the review, each paper had to be related to two themes: LIFU and its therapeutic application to spinal cord-related diseases. LIFU was defined as therapeutic focused ultrasound that modulates neural activity without causing permanent histological damage or significant thermal effect. The inclusion criteria were English language, non-tissue damaging ultrasound neuromodulation, intervention over the spinal cord region, and relation to neuropathic pain or movement disorder (MD). Exclusion criteria were extracorporeal shockwave therapy, tissue destructive ultrasound treatments, non-focused ultrasound, and other topics in ultrasound not related to LIFU. *In vitro* experiments were excluded due to less direct clinical applicability. Reviews, editorials, and conference proceedings were also excluded due to incompatibility with data extraction. The comprehensive list of search terms is included in Supplementary Material A.

### Data extraction and synthesis

For each paper, experimental design data was extracted and summarized in fields of animal species, spinal region, disease model, experimental goal, and outcome measure. Technical aspects of delivered LIFU were also analyzed, with data extraction in the following fields: transducer frequency, PRF, DC, total sonication duration, pulse width, peak acoustic pressure, spatial peak pulse average intensity (*I*_SPPA_), spatial peak temporal average intensity (*I*_SPTA_), power delivered, and temperature change. The results are presented in Tables 1–4 and are discussed further in the following section.

**Table 1:** Sonication parameters of neuropathic pain disease models.

Authors	Target	Frequency (MHz)	PRF (Hz)	Duty cycle (%)	TSP (min)	Pulse width (ms)	Subject	Neuropathic pain model	Outcome
Youn et al. (32)	L5 DRG	–	38^a^	–	3	0.09	SD Rat	Vincristine	Decreased mechanical allodynia (VFF), decreased hyperalgesia (hotplate, Randall-Sellito test)
Prabhala et al. (33)	L5 DRG	11	38	50	3	13	SD Rat	CPNI	Decreased mechanical allodynia (VFF)
Liss et al. (34)	L5 DRG	11	38	50	3	13	SD Rat	CPNI	Decreased mechanical allodynia (longer effect for females), decreased SNAP latency (stronger effect in females)
Hellman et al. (35)	L5 DRG	11	38	50	3	13	SD Rat	CPNI	Decreased mechanical allodynia (VFF), Decreased SNAP latency
Hellman et al. (36)	L5 DRG	11	38	50	3	13	SD Rat	Vincristine	Decreased mechanical allodynia (VFF), decreased hyperalgesia (hotplate)
Hellman et al. (37)	L5 DRG	11	38	50	3	13	SD Rat	CPNI	Decreased mechanical allodynia (VFF, inflammatory cytokine analysis)
Hellman et al. (38)	L5 DRG	2.475	38	50	3.5	13	Yorkshire Farm Pigs	CPNI	Decreased mechanical and thermal allodynia (VFF, heating probe on hind hoof, behavioral testing), decreased SNAP latency and amplitude
Bao et al. (39)	L5 DRG	11	38	50	3	13	SD Rat	CPNI	Decreased mechanical allodynia (VFF), single-unit recordings in the ACC and SI using microelectrodes
Liao et al. (40)	L4-L5	4	800	20	20/day for 4 wk	0.25	SD Rat	CPNI	Decreased mechanical allodynia (VFF), KCC_2_ expression increased

SD rat, Sprague-Dawley rats; SI, somatosensory cortex; ACC, anterior cingulate cortex; CPNI, common peroneal nerve injury.

A dash mark is used when values are not reported.

^a^ The unspecified frequency is reported to be 38 Hz, assumed to be PRF.

**Table 2:** Sonication parameters of MD disease models.

Authors	Target	Frequency (MHz)	PRF (Hz)	Duty cycle (%)	TSP (min)	Pulse width (ms)	Subject	Outcome
Kim et al. (41)	T12	3	1,000	50	3.333	0.5	Mice	Decreased essential tremor
Liao et al. (42)	T8	4	800	50	20/day for 4 weeks	0.625	SD Rat	Decreased spasticity in SCI
Wang et al. (43)	T9	1	800	20	20/day for 4 weeks	0.25	SD Rat	Decreased spasticity in SCI

**Table 3:** Sonication parameters on evoked potential and reflex circuit studied.

Authors	Target	Frequency (MHz)	PRF (Hz)	Duty cycle (%)	TSP (min)	Peak pressure (MPa)	Pulse width (ms)	Subject	Outcome
Kim et al. (41)	T12	3	1,000	50	3.333	1.4	0.5	Mice	No peak-to-peak SSEP difference is seen with electrical stimulation
Kim et al. (41)	L3	3	1,000	50	3.333	2.2	0.5	Mice	No peak-to-peak SSEP difference is seen with electrical stimulation
Tsehay et al. (44)	T11- T12	0.5	1,000	50	5, 10	0.0297	0.5	SD Rat	MEP Suppression for duration of treatment
Liao et al. (45)	L4-L5	4	1,000	20	20	0.5, 1.0, 1.5, 2.0, 2.5, 3.0	0.2	SD Rat	Recruitment of the soleus muscle in a dose dependent manner with increasing peak pressure and *I*_SPTA_
Song et al. (46)	T13, L5 DRG	1.1	2,000	40	1	1.48	0.2	SD Rat	Suppressed H-reflex amplitude and latency Augmented homosynaptic depression Suppressed flexor reflex windup
Song et al. (46)	T13, L5 DRG	1.1	2,000	40	1	0.83, 1.27, 1.72	0.2	SD Rat	Suppressed H-reflex amplitude and latency
Song et al. (46)	T13, L5 DRG	1.1	100	40	1	—	0.2	SD Rat	Minimally suppressed H-reflex latency; minimally increased H-reflex amplitude
Song et al. (46)	T10, S2	1.1	2,000,100	40	1	0.83, 1.27, 1.48, 1.72	0.2	SD Rat	No suppression of H-reflex latency or amplitude

A dash mark is used when values are not reported.

**Table 4:** Studies measuring thermal effects of LIFU and corresponding intensities for authors with at least one reported value of the following: total power delivered, peak pressure, *I*_SPPA_, *I*_SPTA_, and temperature change.

Authors	Total power delivered (W)	Peak pressure (MPa)	*I*_SPPA_ W/cm^2^	*I*_SPTA_ mW/cm^2^	Temperature change (°C)	Safety effects
Youn et al. (32)	—	0.0297	—	—	0.1–1.3	None
Hellman et al. (36)	3, internal sonication	—	—	—	3.21 ± 0.30, 2.34 ± 0.68	None
Hellman et al. (36)	8, external sonication	—	—	—	1.78 ± 0.21, 1.65 ± 0.36	None
Hellman et al. (38)	28	—	—	—	0.80 ± 0.41	None
Kim et al. (41)	—	0.8	9.9	4.95	0.6	None
Kim et al. (41)	—	1.4	28	14	1.6	None
Kim et al. (41)	—	2.2	76	38	3.5	None
Liao et al. (45)	—	3	—	600	<0.025^b^	Spinal cord damage^c^
Song et al. (46)	—	1.48	—	—	6^a^	None

^a^ Song et al. (46) measured the temperature increase in the subcutaneous tissue over the spine rather than the spinal cord itself.

^b^ The temperature changes in Liao et al. (40) were determined through a pre-study and not in the experiment, parameters are unknown.

^c^ Spinal cord damage led to decreased SSEP amplitude, coagulation necrosis, structural destruction, neuron loss and increased inflammatory factors. *I*_SPPA_: spatial-peak pulse-average intensity, *I*_SPTA_: spatial-peak temporal-average intensity. A dash mark is used when values are not reported.

## Results

**Figure 3:**
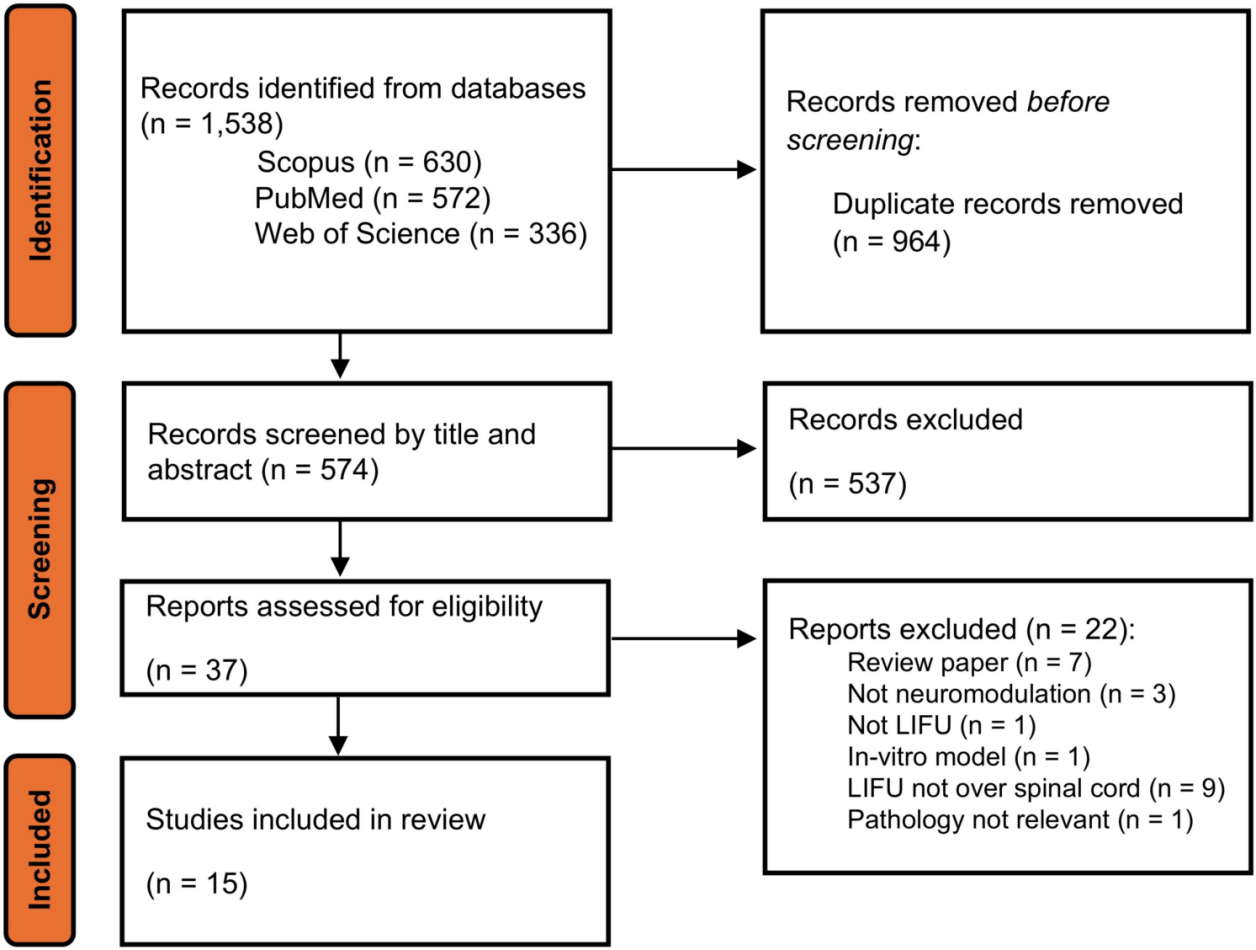
Flowchart of search, screening, and study inclusion.

An initial search resulted in 574 records after the removal of duplicates. These were screened via title and abstract review for relevance and 37 records were selected for full review and assessment. Of these, 15 records met the eligibility criteria to be included in this scoping review (32–46). Figure 3 details our record selection process.

In all records, research was conducted in animals, with 13 studies using rats (32–37, 39, 40, 42–46), one study using swine (38), and one study using mice (41). Studies targeted the spinal cord or dorsal root ganglion of the lower thoracic and lumbar spine, measuring behavioral and functional effects on the hindlimb. Nine studies evaluated the effects of LIFU on an animal model of neuropathic pain (32–38), three used animal models for MD (41–43), and 4 used healthy animals to test the effects on functioning spinal circuits, and sensory and motor pathways (41, 44–46).

### Preclinical efficacy in chronic neuropathic pain models

CNP is currently the most well explored pathology for treatment by LIFU applied to the spinal cord. We identified nine studies that evaluated the effects of LIFU in animal models of neuropathic pain. All studies targeted the lumbar spine region, with eight studies focused on the L5 DRG and one on the L4–L5 spinal cord. Studies used vincristine injection (32, 36), and common peroneal nerve injury (CPNI) (33–40), where the common peroneal nerve is partially ligated with sutures, to induce allodynia in a neuropathic pain model (47). Sensation in neuropathic pain is subjective and difficult to measure using electrophysiology, so behavioral assessments such as Von Frey filament (VFF) testing, for determining mechanical pain threshold, and hotplate testing, for thermal pain threshold, were used to evaluate the effects of treatment in all papers (48). Beyond behavioral testing, two studies incorporated nerve conduction studies to assess LIFU-mediated changes in sensory nerve action potential (SNAP) latency and amplitude, both demonstrating transient improvements in conduction velocity following treatment. As most papers used the same LIFU parameters (Table 1), it was not possible to determine any relationship between parameters and pain modulation.

Youn et al. (32) applied pulsed FUS applied to the L5 DRG of rats with vincristine-induced neuropathy. Compared to untreated rats, those treated with HIFU had significantly increased innocuous and noxious mechanical thresholds, and increased temperature thresholds at both 24 and 48 h after FUS. On histology, pulsed HIFU caused initial cellular swelling, which largely subsided after 24 h, indicating no permanent damage was done.

Prabhala et al. (33) induced chronic peripheral neuropathy using the CPNI model to evaluate the efficacy of LIFU treatment at the L5 DRG. Compared to before treatment, LIFU significantly increased the VFF mechanical withdrawal threshold. After one treatment, the effect remained for 72 h. When the same treatment was applied a week after the first, there were no observed differences in response. No changes were observed for locomotor activity and tissue showed no evidence of damage on histology.

Liss et al. (34) evaluated differences between male and female rats in their response to LIFU treatment at the L5 DRG after CPNI. While both males and females saw an increase in VFF mechanical threshold post-LIFU, the effects lasted seven days for females but only three for males. Thermal threshold improved for both males and females with no difference in response. Additionally, SNAP latency was reduced for 50 min after LIFU in females, but only 25 min in males. With histology, no changes were observed at a total applied dose below 8 W.

Hellman et al. (35) explored the effects of external LIFU targeting the L5 DRG in a rat model of neuropathic pain induced by CPNI. Behavioral assays with VFF testing and hotplate testing confirmed significant increases in both mechanical and thermal thresholds 24 h after a single LIFU treatment, indicating reduced pain sensitivity. Concurrently, nerve conduction studies revealed that LIFU significantly decreased SNAP latencies in both CPNI and sham-CPNI animals for up to 30 min post-treatment with no changes in SNAP amplitude, warranting further investigation into neural circuitry modulation of LIFU mediated pain relief. Histological analysis demonstrated no evidence of neuronal degeneration, confirming tissue safety at the applied dose. This study represents the first *in vivo* investigation of LIFU-induced electrophysiological changes in peripheral nerves and demonstrates that external LIFU can non-invasively modulate DRG activity and provide pain relief in neuropathic rats.

Hellman et al. (36) examined the effects of both internal and external LIFU targeted at the L5 DRG in a rat model of vincristine-induced neuropathy (VIN), a chemotherapy-induced condition characterized by sensory deficits and allodynia. Behavioral testing confirmed successful induction of allodynia using VFF and hot plate tests, with vincristine-treated animals resulting in significantly reduced mechanical and thermal pain thresholds. One LIFU treatment—either internal (2.5 W) or external (8 W)—resulted in significant and sustained reversal of allodynia, with VFF thresholds restored to the pre-treatment baseline for up to 5 days post-treatment. Thermal pain thresholds improved modestly but significantly 24 h post-treatment for both internal and external LIFU. Temperature measurements during treatment produced a peak temperature rise of 3.21 °C and 1.78 °C for internal and external LIFU sonication, confirming a modulatory, rather than ablative, effect. Histological analysis of the L5 DRG revealed no evidence of tissue damage, inflammation, or neuronal degeneration. Importantly, open field testing demonstrated that LIFU did not impair locomotor activity, indicating treatment specificity for sensory modulation.

Hellman et al. (37) expanded upon previous findings by investigating the anti-inflammatory mechanisms underlying the analgesic effects of external LIFU targeted at the L5 DRG in a CPNI rat model. Their study measured cytokine expression in both the DRG and dorsal horn at 24- and 72-h post-treatment, using microarray protein assay and commercial brand Enzyme-Linked Immunosorbent Assay (ELISA) analysis. Following CPNI, animals developed mechanical allodynia, which was reversed by LIFU treatment and sustained for at least 72 h. This allodynia and its reversal were captured not just with behavioral testing in VFF, but also with inflammatory markers. CPNI elevated proinflammatory cytokines TNFa, IL6, IL1b, and CNTF in both DRG and dorsal horn, while LIFU significantly reduced their levels and concurrently increased IL10 expression, particularly at the 72-h mark. IL6 and TNFa changes were detected via ELISA. Additionally, expression of less-characterized inflammatory markers such as SiCAM1, IL3, and thymus-expressed chemokine also shifted in response to LIFU, indicating broader immune modulation.

Hellman et al. (38) conducted the first study to evaluate the feasibility and efficacy of external LIFU targeting the dorsal root ganglion in a large animal model of neuropathic pain. Using a modified LIFU array, the researchers applied treatment to the L5 DRG in swine with neuropathic pain induced by CPNI. In both juvenile (13–14 kg) and larger (∼20 kg) cohorts, LIFU significantly improved mechanical thresholds for up to 5 days and reversed thermal allodynia for at least 24 h. Treatment parameters were optimized in the first cohort and successfully scaled to the second, demonstrating effective DRG modulation at tissue depths up to 4.75 cm. Concurrent nerve conduction studies showed transient increases in conduction velocity and decreases in amplitude following treatment, indicating acute electrophysiological modulation. Importantly, histological analysis staining revealed no signs of cellular damage or degeneration, even in animals that experienced >9 °C spinal cord temperature rise during treatment. While two cases of superficial skin burns occurred early in the study, adjustments to coolant flow and temperature resolved these issues.

Bao et al. (39) measured the effects of LIFU at the L5 DRG on activity in the somatosensory cortex and anterior cingulate cortex, two brain regions implicated in pain sensation, in a rat model of chronic neuropathic pain induced by CPNI. In the somatosensory cortex, LIFU treated animals diverged in their response to treatment, with one group of animals having a robust increase in pyramidal cell spike frequency compared to the non-responding LIFU CPNI, sham-LIFU CPNI, and sham-CPNI groups. In LIFU-responsive animals, pyramidal neuron activity became significantly elevated by 120 min post-treatment and remained heightened for the duration of the 240-min observation period. Interneuron activity in the somatosensory cortex did not exhibit significant changes. In the anterior cingulate cortex, no change was observed in pyramidal neuron activity. The interneuron activity was suppressed compared to the sham-LIFU CPNI group but not the sham-CPNI group, indicating a normalization toward non-pathological activity levels.

Liao et al. (40) investigated neuropathic pain in the context of KCC_2_, a pro-inhibitory K-Cl channel that reduces neural excitability. The researchers hypothesized that LIFU inhibits the activation of regulatory proteins, CaMKIV and p-CREB, which reduces the down regulatory effect on KCC_2_, leading to reduced pain perception. The researchers evaluated the effects of repeated LIFU treatment at L4–L5 on the expression of these proteins in rats with CPNI. After 3 weeks of treatment, the mechanical threshold for allodynic response was lowered in both CPNI groups but higher in the group receiving 20 min/day LIFU. After 4 weeks of treatment, rats treated with LIFU had increased expression of KCC_2_ and decreased expression of CaMKIV and p-CREB in the L4–L5 spinal cord compared to the untreated CPNI group. No histological evidence of tissue damage was observed.

### Preclinical efficacy in SCI and MD models

Three studies evaluated the efficacy of LIFU in treating symptoms in MD models, including two studies on spasticity and hyperreflexia after spinal cord injury and one study on attenuation of essential tremor. Overall, LIFU was found to have an inhibitory effect on spinal circuits associated with hyperreflexia and tremor. Both online effects (41) and long-term offline effects (42, 43) were observed, suggesting the involvement of multiple mechanisms. Table 2 details the LIFU parameters used to achieve these effects. The association between parameters and effects on tremor or spasticity could not be determined due to limited studies.

Kim et al. (41) studied mice with harmaline-induced essential tremor and found applying LIFU to T12 of the spine effectively reduced hindlimb tremor activity as measured by EMG. They did not observe depression of motor evoked potential (MEP) or somatosensory evoked potential (SSEP) signals under sonication, indicating that LIFU was selective in its effects on the spinal tracts.

Liao et al. (42) assessed whether repeated treatments with LIFU can reduce SCI-related spasticity. The study hypothesized that LIFU treatment would reduce spasticity by inducing KCC_2_ expression, a neuronal K-Cl cotransporter that is normally downregulated in the spinal cord below the level of injury leading to excess motor neuron excitability. Rats with complete spinal cord transection treated with 20 min/day LIFU over 4 weeks were compared to those that did not receive LIFU treatment. At 4 weeks post-injury, rats treated with LIFU showed a greater threshold of mechanical stimulation, greater locomotor function as demonstrated by higher Basso, Beattie, and Bresnahan score, reduced spasticity as measured by frequency-dependent depression of H-reflex, and shorter EMG response time than rats with SCI not treated with LIFU. Compared to both healthy rats and those with SCI treated with LIFU, untreated rats with SCI had significantly decreased expression of KCC_2_, offering support for this potential mechanism mediating the long-term effects of LIFU. Additionally, the study found that LIFU treatment can directly activate neurons as found by a detectable EMG response during sonication.

Wang et al. (43) also tested the long-term effects of repeated LIFU treatment on rats with complete spinal cord transection. The treatment group was treated with LIFU for 20 min/day for 4 weeks and compared to a non-treatment SCI group and a healthy, sham group. At 4 weeks, rats treated with LIFU had a higher mechanical threshold for spastic reaction compared to the non-treatment SCI group, though both groups had far lower mechanical thresholds than healthy rats. Bioinformatic analysis revealed significantly higher expression of growth associated protein 43 (Gap43) in SCI rats not treated with LIFU, implicating the overexpression of this protein as a potential mechanism of spasticity development reversed by LIFU.

### Effects on evoked potentials and reflex circuits in healthy animal models

Four studies investigated the effects of LIFU on spinal reflexes and motor pathways in healthy rodents, providing insight into the potential neuromodulation mechanisms relevant to clinical applications. Three studies evaluated the effects on MEPs (41, 42, 44), demonstrating both suppression and stimulation of signals depending on sonication parameters and location. One study found suppression of spinal reflexes with dependence on dose, location, and waveform parameters (46). Study outcomes and sonication parameters are summarized in Table 3.

Song et al. (46) studied the effects of LIFU on spinal reflexes in healthy rats. The study demonstrated that LIFU on the spine can reversibly suppress monosynaptic (H-reflex) and polysynaptic (flexor and hotplate withdrawal) spinal reflexes. H-reflex amplitude was depressed in a pressure-dependent manner and with stronger depression with treatment at the motor neuron level during LIFU sonication of the motor neuron region or dorsal root ganglion of the sciatic nerve. Further, an inhibitory effect was observed with a pulse repetition frequency of 2 kHz while a smaller excitatory effect was observed with PRF 100 Hz. LIFU treatment selectively inhibited C-fiber-mediated windup in flexor reflex circuits and increased the latency of withdrawal responses in awake animals exposed to thermal stimuli. The researchers determined that the minimal tissue heating could not explain the observed effects. No tissue damage was observed with the histology of the treated segments.

Tsehay et al. (44) demonstrated full suppression of motor evoked potentials by LIFU of the rat spinal cord at T10–T12. The suppression of hindlimb MEPs was reversible, with gradual recovery after stopping LIFU. There was no difference in recovery time among the groups, regardless of the total LIFU sonication period. The researchers found no evidence of spinal cord damage or inflammation on histological analysis and quantitative polymerase chain reaction.

In mice, Kim et al. (41) found inhibition of MEPs when LIFU was applied at T12 but facilitation of MEPs when LIFU was applied at L3. The inhibitory effects that were observed exhibited a dose dependent relationship with LIFU pressure from 0.8 MPa to 2.2 MPa. Both inhibitory and enhancing effects were rapidly observed after beginning LIFU sonication. The time to return to baseline after treatment ceased was longer for inhibitory than stimulatory effects, indicating different mechanisms mediating each effect. Somatosensory evoked potentials in mice were not affected by LIFU applied at either spinal level. LIFU was found to not directly stimulate MEP or SSEP signals.

Liao et al. (45) evaluated the effects of LIFU on MEP and expression of neuronal activation markers in healthy rats. Applying LIFU over the L4–L5 vertebrae activated motor neurons and recruited the soleus muscle in a pressure-dependent manner from 0.5 MPa to 3.0 MPa. Protein c-Fos, a marker of neural activity, and GAD65, a marker of GABAergic synaptic activity, were both elevated, with 1.5 MPa acoustic pressure eliciting the greatest increase in expression. Signs of spinal cord injury were observed with 3.0 MPa acoustic pressure, including decreased SSEPs, neuronal loss, inflammation, and increased apoptotic markers. Tissue injury was not observed at acoustic pressures of 1.5 MPa or less.

### Safety

Safety is a critical consideration for LIFU, as the application of focused acoustic waves at high enough power or over a long enough duration has the potential to heat tissue and lead to permanent damage. From the articles identified, eleven evaluated the safety of LIFU, with nine reporting that treatment did not cause detectable swelling or damage (32–36, 38, 40, 41, 44–46), Youn et al. (32) found that LIFU treatment caused initial cellular swelling, which could be observed on histology. This largely subsided after 24 h, indicating no permanent damage was done, with full recovery of healthy cellular structure by five days after treatment. Liao et al. (45) observed decreased SSEP amplitude, neuronal loss, and increased expression of inflammatory markers IL1-B and TNF-a at 3.0 MPa acoustic pressure but not for any groups treated with 1.5 MPa or lower acoustic pressure. The higher power necessitated by a larger swine model and deeper spinal structures in Hellman et al. (38) caused superficial skin burns during sonication. The researchers implemented a water-cooling system to prevent future burns. The temperature changes in the treated region were measured in five studies, detailed in Table 4. Temperature change is positively correlated with increased *I*_SPTA_, as observed in the identified studies. However, incomplete reporting of sonication parameters limits a clear understanding of what is a nondestructive sonication parameter.

## Discussion

LIFU resulted in online and offline effects in our literature. The neuromodulation effects observed during sonication are known as the online effects of LIFU. The persisting biological and behavioral changes observed after cessation of sonication are known as the offline effect of LIFU. We discuss the potential gaps in literature regarding the observed online and offline effects of LIFU. Further, we report discrepancies in reporting parameters and commonly used nomenclature for LIFU studies.

### LIFU online effects

The online effects of LIFU, can be both excitatory and suppressive. While the exact mechanism of excitation and suppression remains unclear, FUS parameters and target regions likely influence the modulation type (49). In our review, only one study, Liao et al. (45), reported excitatory online effects, demonstrating a linear correlation between peak acoustic pressure and EMG-measured activation of the soleus muscle. All other studies focused on inhibitory effects, but whether these result from activation of inhibitory circuits or direct neuronal suppression remains uncertain. A possible frequency and dose-dependent effect across spinal tracts may also contribute to variability.

LIFU is generally believed to exert its effects through non-thermal and non-cavitation mechanisms. Liss et al. (34), Bao et al. (39), and Youn et al. (32) suggested that activation of thermosensitive ion channels two pore potassium channel family (K2P) and voltage gated sodium channel (Na_V_) caused hyperpolarization, reducing neuronal excitability. Increased conductance of Na_V_ channel in heated tissue has been hypothesized to be a driver of LIFU stimulatory effect (50), though its role in inhibition has been contested (51). It is difficult to map potential mechanisms to specific study findings because only six out of our 15 papers reported any temperature data of the targeted tissue.

Conversely, Tsehay et al. (44) observed a 3–4 min delay before maximal MEP suppression, a pattern typically associated with thermal mechanisms. Yet, MEP suppression ceased immediately after sonication, with a slight spinal cord temperature elevation (0.3–1°C above baseline). This suggests that thermal effects are unlikely the primary cause of MEP suppression, warranting further exploration to characterize the mechanism and change of MEP under LIFU.

### LIFU offline effects

The offline effects of LIFU refer to the persisting biological and behavioral changes observed after cessation of LIFU. The specific changes and mechanisms of these offline effects are not well understood. All studies included in this review reported minimal to no histological damage after LIFU, thus suggesting that these offline effects are likely due to a combination of induced neuroplasticity and changes at the proteomic, metabolomic, and transcriptomic scales.

Studies examining CNP mainly reported offline effects lasting between 24-h and 7-days in CNP models induced by CPNI (*n* = 6) or vincristine (*n* = 2) after one or two sonication trials within the same day with the exception of Liao et al. (40). However, the offline effects were not uniform between different behavioral tests. Thermal hyperalgesia modulation seemed to be the least affected, lasting 48-h in Youn et al. (32), 24-h in Hellman et al. (37), and 24-h in Hellman et al. (38). In comparison, mechanical nociceptive suppression exhibited a more varied timeframe. Youn et al. (32), Hellman et al. (36), and Hellman et al. (38) reported offline effects on increased mechanical thresholds as tested by VFF on CNP models up to five days after LIFU. Liss et al. (34) reported suppression of mechanical allodynia in female rats for up to seven days and Hellman et al. (35) reported a 30-min suppressive effect on mechanical allodynia. Bao et al. (39) only recorded allodynic effects for 240-min post sonication due to the study design, making the exact duration of the offline effects impossible to discern.

These findings contrast with findings by Kim et al. (41) and Tsehay et al. (44) where the removal of LIFU caused a return to baseline on much shorter timescales. Kim et al. (41) found that harmaline-induced essential tremor returned to baseline tremor characteristics 50-s after the cessation of LIFU sonication. Tsehay et al. (44) observed MEP responses returning to baseline within 5-min post-treatment, with approximately 80% recovery by 10-min in a non-pathological rat model, suggesting a dose-dependence in offline duration effect. These relationships were also observed in Liao et al. (40) where the VFF threshold increased as the treatment progressed through four weeks. Although the two studies may be difficult to directly compare, it presents a need for an investigation into the effect of temporal peak intensity of LIFU on the duration of its offline effect.

Differences in disease models, sonication parameters, and targeted anatomical sites may explain the variability in offline outcomes. Notably, CNP-focused studies employed relatively consistent LIFU parameters, characterized by lower PRF, higher duty cycles, and longer pulse durations, compared to studies targeting motor function as seen in Kim et al. (41) and Tsehay et al. (44). It is unclear if the difference in offline effects were due to the different parameters used, tissue specific response to LIFU, or a combination of both factors.

Thus, there is a growing trend to better understand and characterize the mechanism of neuroplastic change induced by LIFU. Previous studies of disconnected neurons found that LIFU modulated cortical neurons independent of thermal dose without observable cavitation (13). However, similar studies have not been conducted in spinal tissue and the expected spinal cord and DRG tissue response to LIFU is unclear. We identified five papers that analyzed various histological and biochemical features to propose an explanation for the offline behavior of sonicated neural tissue.

Youn et al. (32) reported cellular edema in the sonicated DRG region which resolved 48-h post-LIFU. This histological feature was accompanied by a slight increase in the number of hyaline inclusions and increased satellite cells. The cellular edema expanding around the necrotic ablated core was believed to contribute to delayed onset side-effects of HIFU thalamotomy, such as ataxia and dysarthria. These symptoms typically resolved when edema dissipated (52). While cellular edema may be a primary driver of offline neuromodulation, the exact temporal dynamic of the observed cellular edema and its relationship to the near-linear degradation of offline CNP suppressive effect is unclear. No subsequent studies in our review found similar edematous features, highlighting the need for further studies to better understand this relationship.

Hellman et al. (37) saw long-lasting shifts in DRG and dorsal horn cytokine levels after CPNI and subsequent LIFU. The authors explored many markers, the most notable of which are TNFa, IL6, CNTF, IL1b, TIMP1, and IL-10. The TNFa-IL6-CNTF axis has been implicated in peripheral pain cascades (53). IL1b is upregulated in neuropathic pain models (52). TIMP1 and IL-10 have been linked to TNFa expression (53). CPNI elevated TNFa, IL6, IL1b, CNTF, TIMP1, and decreased IL-10. Strikingly, LIFU reversed this cytokine shift at both timepoints, reducing TNFa, IL6, IL1b, CNTF, and TIMP1 while increasing IL-10. This study is the first-time cytokine profiling has been explored in CPNI LIFU models, offering a possible explanation of how LIFU modulates pain.

Liao et al. (40, 45) and Wang et al. (43) examined the changes under daily LIFU over a four-week total sonication period. In these studies, rodent behavioral changes persisted between LIFU sonication sessions and at final post-treatment measurements. Liao et al. (42) and Wang et al. (43) observed that rats in the LIFU positive groups maintained improved spasticity thresholds while Liao et al. (40) observed persistent neuropathic pain relief. Underlying these behavioral changes, these studies also demonstrated sustained changes in several molecular markers. In Liao et al. (40), decreased regulatory proteins CaMKIV and p-CREB were measured after weeks of treatment, indicating the restoration of the baseline KCC_2_ pathway. Indeed, in both Liao et al. studies (40, 45), the upregulation of KCC_2_ four weeks post-treatment suggest the sustained restoration of GABAergic inhibition in spinal neurons (54). CaMKIV and p-CREB, key regulators of transcription and of KCC_2_, were also explored and found to be downregulated in Liao et al. (40). The long-term upregulation of KCC_2_ and downregulation of CaMKIV and p-CREB suggests lasting transcriptional reprogramming initiated by LIFU treatment. Wang et al. (43) also conducted a proteomic analysis, observing changes four weeks post-treatment with downregulation of GAP43, a marker of axon growth and excitability. Although this finding seems paradoxical to improved spasticity, the authors explained that neural reorganization may be responsible for the development of spasticity. Thus, a lower GAP43 indicates inhibition of spasticity. Together, these results indicate a relationship between LIFU treatment and induction of proteomic and transcriptional changes. Although the causative mechanism was not investigated, these findings suggest the potential for long-term and therapeutic neuromodulation through LIFU treatment.

### Inflammation and neuropathic pain

The development of neuropathic pain is closely tied to inflammatory processes (55, 56). Hellman et al. (37) and Liao et al. (40) explored this neuropathic pain-inflammation axis using LIFU. While both studies show reduced inflammatory markers and pain levels as a result of LIFU stimulation, the exact mechanism of how LIFU downregulates this inflammation was not described. In a 2017 review of LIFU-induced immunosuppression, Yang et al. (57) hypothesized that LIFU upregulates several anti-inflammatory genes and mediates immunosuppressive signaling through exosome transport to neighboring cells. While the anti-inflammatory effect of LIFU is well demonstrated, a definite consensus on its mechanism has yet to be reached.

### Sites of stimulation for CNP: dorsal root ganglia and the spinal cord

Inhibition of neuropathic pain through traditional spinal region electrical stimulation is typically conducted at two common targets: the dorsal root ganglia and the dorsal horn of the spinal cord (58, 59). Although these two sites are relatively close together and encapsulated in the dura, a key difference is that the dorsal horn is part of the central nervous system while the DRG is part of the peripheral nervous system and lies lateral to the spinal cord in the neural foramina. The DRG is made up of the soma of pseudo-bipolar neurons, serving as a critical relay point between the peripheral and central nervous systems (58). While SCS has been more extensively studied compared to DRG electrical stimulation, it may be less effective in managing focal pain syndromes (e.g., complex regional pain syndrome, phantom limb pain) and a common cause of explanation is loss of therapeutic efficacy over time (25, 60). While the exact mechanism of DRG stimulation is still a topic of ongoing research, it is established that pain pathways are modulated differently between SCS and DRG electrical stimulation (61). Thus, DRG offers an alternate mechanism of pain inhibition in order to capture a larger population of chronic pain patients, such as those inadequately managed through SCS (25). There is clinical evidence supporting the use of DRG electrical stimulation as salvage therapy for patients who lost therapeutic benefit from SCS (62).

This review identified one study (40) that targeted the spinal cord and eight studies (32–39) that took inspiration from the success of DRG electrical stimulation to target the DRG using LIFU for CNP. Because of the small sample size, variations in study protocol, and different outcome measurements, the relative efficacy of LIFU sonicating the two target regions cannot be directly compared. Further, there is a missing understanding of the mechanism of action for LIFU in pain modulation and its relation to existing pain theories (63). More research is required to explore the effects of LIFU on DRG and specific targets within the spinal cord, and their mechanism of pain modulation.

### Sex-specific effects

Liss et al. (34) found greater nerve conduction latency and reduced mechanical allodynia improvement in female rats compared to male rats treated with LIFU post-CPNI when assessed by VFF and behavioral analysis. These findings are consistent with emerging evidence pointing to sex-specific differences in pain processing pathways (64–66). Previous studies found that biological females typically have lower heat and pressure pain thresholds and tolerances and increased temporal summation of pain than biological males (64, 67). Some studies suggest that pain perception may be influenced by hormone-modulated immune responses (64, 68).

While there is literature comparing of sex-dependent effect of electrical neuromodulation using SCS (69), Liss et al. (34) is the only study identified in this scoping review to study the sex-dependent effect of LIFU. All other studies identified in this scoping review exclusively used male animals. Our review highlights the need for further studies analyzing sex-dependent responses to LIFU sonication, especially when considering the increased prevalence of CNP among the population of biological females (64, 70–72).

### Need for standardization of reporting parameters

There is limited variation in the parameters used in spinal cord neuromodulation experiments, with research groups often reusing the same parameters as their previous publication. Technical explanations of ultrasound device design are also underexplored or missing. Consequently, the reporting of FUS sonication parameters remains unstandardized across research groups, hindering meaningful comparisons between studies. The recommendations for reporting therapeutic ultrasound treatment parameters, as outlined by Padilla and ter Haar (73), specify the minimum reporting information to be frequency, scanning regimen, pulse length, PRF, acoustic power as a function of voltage to the transducer, *I*_SPTA_ of the target site, *I*_SPPA_ or mechanical index, uncertainty of measurement, and estimate of *in situ* values. Only one out of fifteen studies that were reviewed reported all of these recommended parameters. Especially in the growing field of LIFU, establishing standardized reporting practices would enhance the reproducibility of future investigations and improve the comparability of results within the existing literature.

### Need for standardization of nomenclature

Youn et al. (32) was included in this literature review despite self-labeling as HIFU, or high intensity focused ultrasound, study. We justify its inclusion because of its nondestructive nature and the use of largely similar parameters and methods to the subsequent papers labeled LIFU (36). The difference between the currently utilized definition of HIFU and LIFU is unclear. Many studies characterize the two based on outcomes, such as thermal tissue damage, while others use sonication parameters. However, the use of this terminology can get extremely nuanced. A temporary thermal lesion created using HIFU parameters used for targeting of FUS thalamotomy ablation therapies is not considered LIFU in any literature; while LIFU that causes tissue damage, such as one found in Liao et al. (45), is classified as LIFU. In this review, LIFU was broadly defined as therapeutic focused ultrasound that modulates neural activity without causing permanent histological damage or significant thermal effect. Further complicating the matter, LIFU was frequently described with different acronyms such as LiFUS, FUS, tsFUS, and LiUS.

## Conclusion

The use of LIFU in the spinal region for CNP and MD is a new and exciting development towards noninvasive and precision medicine to improve patient qualities of daily life. We have identified many gaps in literature including the lack of parameter optimization, inconsistent treatment effects, and unclear mechanism of online and offline LIFU neuromodulation in the spine region. Further characterization of LIFU in animals is needed to address the gaps in knowledge.
