# Supplementary material for: Low-intensity focused ultrasound of the spine in the treatment of chronic pain and movement disorder: a scoping review
Source: Front Pain Res (Lausanne). 2025 Jun 17;6:1606672. doi: 10.3389/fpain.2025.1606672 (PMC12209223; doi:10.3389/fpain.2025.1606672)
Supplement: Supplementary file 1 [file Datasheet1.docx]

Supplementary Data

**Supplement A:** Search query for indexed databases, 12/17/24 -

**PubMed**

("Spinal Cord"[tiab] OR "Dorsal Root Ganglion"[tiab] OR "Spinal Cord"[MeSH] OR neuropathic pain[tiab] OR chronic pain[MeSH] OR chronic pain [tiab] OR low back pain [tiab] OR low back pain [MeSH] OR hyperalgesia[tiab] OR allodynia[tiab] OR neuralgia[MeSH] OR neuralgia[tiab] OR paroxysmal pain[tiab] OR algesia[tiab] OR peripheral nerve pain[tiab] OR peripheral nerve injuries[MeSH] OR peripheral nerve injur*[tiab] OR lower back pain[tiab] OR idiopathic pain[tiab] OR complex regional pain syndrome[MeSH] OR complex regional pain syndrome[tiab] OR diabetic neuropath* [tw] OR diabetic neuropathies [MESH])

AND

("Low-Intensity Ultrasound"[tiab] OR "Focused Ultrasound"[tiab] OR "Pulsed Ultrasound"[tiab] OR "LIFU"[tw] OR "tsFUS"[tw] OR "Ultrasonic waves"[MeSH] OR "Ultrasonic therapy"[MeSH])

**Web of Science:**

TS=("Spinal Cord" OR "Dorsal Root Ganglion" OR "neuropathic pain" OR "chronic pain" OR "low back pain"

OR "hyperalgesia" OR "allodynia" OR "neuralgia" OR "paroxysmal pain" OR "algesia" OR "peripheral nerve pain"

OR "peripheral nerve injur*" OR "lower back pain" OR "idiopathic pain" OR "complex regional pain syndrome"

OR "diabetic neuropath*" OR "diabetic neuropathies")

AND

TS=("Low-Intensity Ultrasound" OR "Focused Ultrasound" OR "Pulsed Ultrasound" OR "LIFU" OR "tsFUS"

OR "Ultrasonic waves" OR "Ultrasonic therapy")

NOT

TS=("Extracorporeal Shockwave Therapy" OR "ESWT" OR "Shockwave Therapy")

**Scopus:**

TITLE-ABS-KEY("Spinal Cord" OR "Dorsal Root Ganglion" OR "neuropathic pain" OR "chronic pain"

OR "low back pain" OR "hyperalgesia" OR "allodynia" OR "neuralgia" OR "paroxysmal pain"

OR "algesia" OR "peripheral nerve pain" OR "peripheral nerve injur*" OR "lower back pain"

OR "idiopathic pain" OR "complex regional pain syndrome" OR "diabetic neuropath*" OR "diabetic neuropathies")

AND

TITLE-ABS-KEY("Low-Intensity Ultrasound" OR "Focused Ultrasound" OR "Pulsed Ultrasound"

OR "LIFU" OR "tsFUS" OR "Ultrasonic waves" OR "Ultrasonic therapy")

AND NOT

TITLE-ABS-KEY("Extracorporeal Shockwave Therapy" OR "ESWT" OR "Shockwave Therapy")
